# Investigating the Impact of Host Genetics on the Risk of Disease Progression in Individuals With Influenza

**DOI:** 10.1002/iid3.70394

**Published:** 2026-03-11

**Authors:** Sara Bohnstedt Mørup, Maja Milojevic, Seyed Mahmood Taghavi Shahri, Brad T. Sherman, Weizhong Chang, Ruth Lynfield, Marie Helleberg, Melissa Skeans, Lars Østergaard, Line Borgwardt, Norman Gerry, Marcelo Losso, Kenneth Baillie, Richard Davey, Dominic E. Dwyer, Chansavath Phetsouphanh, Vasileios Papastamopoulos, Richard M. Novak, Daniel D. Murray, Joanne Reekie, Jens D. Lundgren, H. Clifford Lane, Cavan Reilly, For the INSIGHT FLU 002 Plus and FLU 003 Plus Study Groups

**Affiliations:** ^1^ Centre of Excellence for Health Immunity and Infections (CHIP) and INSIGHT Copenhagen ICC Copenhagen Denmark; ^2^ Laboratory of Human Retrovirology and lmmunoinformatics Frederick National Laboratory for Cancer Research Frederick Maryland USA; ^3^ Minnesota Department of Health Saint Paul Minnesota USA; ^4^ Department of Infectious Diseases Copenhagen University Hospital, Rigshospitalet Copenhagen Denmark; ^5^ Department of Clinical Medicine University of Copenhagen Copenhagen Denmark; ^6^ Division of Biostatistics, School of Public Health University of Minnesota Minneapolis Minnesota USA; ^7^ Department of Infectious Diseases Aarhus University Hospital Aarhus Denmark; ^8^ Department of Clinical Medicine Aarhus University Aarhus Denmark; ^9^ Center for Genomic Medicine, Copenhagen University Hospital Rigshospitalet Copenhagen Denmark; ^10^ Advanced Biomedical Labs (ABML) Cinnaminson New Jersey USA; ^11^ Hospital General de Agudos JM Ramos Mejia Buenos Aires Argentina; ^12^ Baillie Gifford Pandemic Science Hub, Centre for Inflammation Research University of Edinburgh Edinburgh UK; ^13^ National Institute of Allergy and Infectious Diseases Bethesda Maryland USA; ^14^ Department of Virology, Centre for Infectious Diseases and Microbiology Westmead Hospital and University of Sydney Westmead New South Wales Australia; ^15^ The Kirby Institute, UNSW Sydney Australia; ^16^ Evangelismos Hospital Athens Greece; ^17^ Section of Infectious Diseases University of Illinois at Chicago Chicago Illinois USA

**Keywords:** GWAS, host genetics, severe influenza, SNP‐microarray, targeted analyses

## Abstract

**Background:**

Knowledge of the human genetic contribution to the risk of complications from influenza is limited. This study assessed the association between human single‐nucleotide polymorphisms (SNPs) and disease progression in individuals with influenza.

**Methods:**

A targeted analysis of 10 SNPs with prior evidence in COVID‐19 and a genome‐wide association study (GWAS) were used to assess associations between SNPs and disease progression in two multinational cohorts with suspected or laboratory‐confirmed influenza: a hospitalized cohort (*n* = 1634) and a pooled cohort of hospitalized and outpatients (*n* = 3469). Disease progression was defined as prolonged hospitalization (> 28 days), progression to mechanical ventilation, admittance to intensive care unit, or death (for hospitalized individuals) or progression to hospitalization or death (for outpatients).

**Results:**

Disease progression was observed in 9.1% of hospitalized patients and 2.2% of outpatients. Age was a significant risk factor for disease progression, with 20% increased odds of disease progression per 10‐year increase in age (OR: 1.20, 95%CI: 1.08–1.33, *p* < 0.001). Disease progression rates also differed by continent (*p* < 0.0001). Targeted SNP analyses did not identify significant associations with disease progression; however, the strength of associations was most pronounced in sensitivity analyses for the pooled cohort in individuals < 65 years old. GWAS analyses did not identify significant common SNP associations in either the hospitalized or pooled cohorts, nor in sensitivity analysis of (1) individuals with laboratory‐confirmed influenza and (2) those aged < 65 years.

**Conclusion:**

In a geographically diverse cohort of individuals with influenza, the genetic links to disease progression only started to become evident in the sensitivity analyses, mainly when looking at younger individuals. The power to detect associations was limited by the rate of disease progression and heterogeneity in phenotypes of the individuals studied, and therefore, additional studies focused on the role of genetics in influenza disease progression are needed.

## Graphical abstract

We utilized a novel targeted and genome‐wide association study (GWAS) approach, whereby SNPs with prior evidence in other viral upper respiratory tract infections were assessed in a targeted approach and all other SNPs in a GWAS approach. The probability of making a Type 1 error (i.e., the “alpha”) was split between the targeted analysis and the GWAS, which, even after appropriate correction for multiple testing, allowed for a less conservative investigation of SNPs with reasonable prior evidence. Despite this, we did not detect any significant associations with SNPs and disease progression when we performed the targeted and GWAS analyses in a cohort of hospitalized, or a pooled cohort of hospitalized and outpatients with known or suspected influenza.

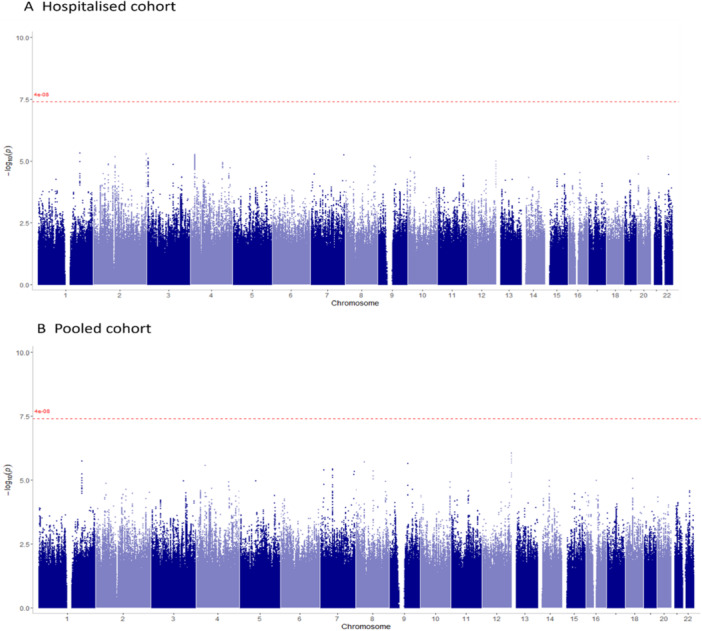

## 1 Introduction

While most influenza and other viral upper respiratory tract infections (vURTI) in humans cause only mild if any symptoms [1], in some cases, influenza can lead to significant morbidity and mortality in children and adults [2]. Complications include secondary infections, particularly bacterial pneumonia, and the exacerbation of underlying chronic conditions. Influenza can also result in severe systemic inflammatory responses causing acute respiratory distress syndrome or death. Several well‐known, nongenetic host risk factors for severe influenza have been identified, including extremes of age, medical comorbidities, immune function, obesity, and pregnancy [3, 4]. Additionally, the pathogenicity of the viral strain as well as the viral load present at the time of infection can affect the outcome of vURTI [1]. Furthermore, environmental factors, including the availability of standard‐of‐care medical interventions for organ support and antiviral medication, also impact outcome. However, severe complications are often observed in otherwise healthy adults without any of the aforementioned risk factors and are suspected to be caused by host genetic factors [3, 5, 6, 7, 8, 9], including variation in genes affecting the immune response [10]. Studies have shown that a proportion of individuals developing life‐threatening influenza infection have inborn errors of immunity (IEIs) caused by rare monogenic pathogenic variants, including defects of interferon‐mediated immunity, critical for the antiviral immune response [3, 11, 12, 13, 14, 15, 16]. Current knowledge of the host genetics´ role in the development of severe influenza has primarily been derived from genome‐wide association study (GWAS) [17, 18, 19]. Genetic variants have been reported within genes functioning in immune response to infection, including the intracellular control of viral replication (*IFITM3, TMPRS22*) and defective interferon responses (*GLDC, IRF7, IRF9*), including mechanisms which may explain the prolonged viral replication in individuals with severe influenza [1]. However, these studies have been limited by size [19, 20] and have shown inconsistent results. Additionally, in a systematic review and meta‐analysis investigating the contribution of host genetic variation to severe influenza, which included 34 human genetic association studies and investigated 20 different genes, only one single‐nucleotide polymorphism (SNP), rs12252‐C in the Interferon Induced Transmembrane Protein 3 (*IFITM3*) gene, showed a significant association, where different populations had opposite effects [4]. In particular, homozygosity of rs12252‐C, which leads to impaired intracellular control of viral replication and has been associated with severe influenza [1, 21]. The rs12252‐C association with severe influenza has predominately been observed in the Han Chinese population, where the SNP is common [22]. However, an excess frequency of this SNP has also been reported in Caucasians hospitalized with influenza [23].

More recently, GWASs in people with SARS‐CoV‐2 infection detected several genome‐wide significant SNPs associated with individuals' risk of developing severe COVID‐19, including SNPs located within the Interferon pathways [24]. SNPs associated with severe COVID‐19 may also affect progression of influenza due to shared immune‐genetic pathways. By studying these mechanisms and through the discovery of other genetic risk factors of severe Influenza, novel immunological mechanisms may be identified that can help identify those at greatest risk of severe disease or as targets for therapeutic intervention. As prior host genetic studies of risk for severe influenza have generally been performed in small, single‐center cohorts, this study aimed to investigate the relationship between host genotypes and risk of disease progression in a multi‐ethnic cohort of individuals with known or suspected influenza through a GWAS, as well as targeted analyses of SNPs with prior evidence in genetic studies of vURTIs.

## 2 Methods

### 2.1 Patient Population

This study investigated adults (age ≥ 18 years) from the INSIGHT FLU 002 Plus and INSIGHT FLU 003 Plus observational cohorts, which recruited outpatients (FLU 002 Plus) or hospitalized (FLU 003 Plus) individuals with known or suspected influenza across Europe, North and South America, Asia, and Australia [25, 26, 27]. Both studies were initiated in response to the influenza A/H1N1 pandemic in 2009 and were continued to monitor and obtain clinical data and biological samples from individuals infected with viral respiratory pathogens. In the FLU 002 Plus (outpatients) study, inclusion required suspected influenza infection, whereas for FLU 003 Plus (hospitalized), inclusion required a more stringent local diagnosis. However, both cohorts collected data on any locally performed polymerase chain reaction (PCR) testing for influenza and had central laboratory performed influenza PCR testing on collected respiratory tract samples.

Our study included a subgroup of participants from these two cohorts, who had given consent for comprehensive genetic analysis. The primary analysis focused on the hospitalized cohort, which consisted of all genotyped individuals from the FLU 003 Plus study and defined disease progression as prolonged hospitalization, progression to intensive care unit (ICU) admission and/or mechanical ventilation (MV), or death assessed at day 28 after enrollment.

Additionally, a secondary analysis was performed on a pooled cohort, which, in line with prior work, combined FLU 002 Plus and FLU 003 Plus [26]. However, as an influenza diagnosis was not required for enrollment into FLU 002 Plus, to better align the two cohorts, the pooled cohort consisted of all genotyped individuals in FLU 003 Plus (hospitalized) and genotyped individuals in FLU 002 Plus (outpatients) with either local or central laboratory confirmed influenza. For the pooled cohort, the outcome was disease progression assessed at day 28 and defined as either: prolonged hospitalization, ICU admission and/or progression to MV, or death (for hospitalized individuals) or progression to hospitalization or death (for outpatients).

### 2.2 Study Cohort Genotype Data and Quality Control (QC)

The study cohort data and QC, as well as imputation, genetic‐based ancestry assignments based on ADMIXTURE and principal components analysis (PCA), and removal of outliers are described in supplementary methods.

### 2.3 Statistical Analysis

To identify the key nongenetic covariates to include in the genetic association analyses, first the effect of potential nongenetic risk factors on disease progression as a binary outcome at day 28 were evaluated using multivariable logistic models, selected for compatibility with standard GWAS model options, straightforward interpretation of the output, and consistency with analytical approaches in previous FLU 002 and FLU 003 studies [26]. Basic model assumptions were assessed before analyses and adjustments were made where necessary (e.g., outlier removal and replacement or exclusion of collinear variables). Sex, baseline age, and continent were included as main predictors in the nongenetic model. To identify additional independent risk factors, the effect of adding an additional factor to the base model was evaluated for the following factors: smoking status, body mass index, self‐reported race, flu type, pregnancy (in the subset of women aged 18–45 years), and the presence of co‐morbidities [26]. The specific co‐morbidity categories to assess were decided a priori, selected based on the key comorbidities investigated in a previous study of FLU 002 and FLU 003 participants by Lynfield et al. [26]. These comorbidities are mainly conditions affecting respiratory functions or contributing to immune dysfunction or a compromised immune state, and therefore highly relevant to influenza outcomes. The Wald method was used for testing continuous and binary factors, and the Likelihood ratio method was employed for testing predictors with more than two levels. A *p*‐value less than 0.05 was deemed statistically significant unless otherwise specified.

We used multivariable logistic regression to assess SNP associations with disease progression in both the targeted and GWAS analysis (see supplementary methods for GWAS details). Models were adjusted for potential confounders based on the variables in the main nongenetics model, specifically age and sex. However, given that the standard genetic analysis adjustments for population structure and continent add redundant information to the model, continent was excluded as a variable. Instead, principal components 1–5 were used to represent differences at the continent‐level in genetic ancestry. PCA eigenvalues show that the first five principal components explain over 95% of the genetic variation in the combined study population and reference population, and visualization of the pairwise principal component plots confirmed that the first five principal components captured intercontinental genetic differences.

### 2.4 Selection of SNPs for the Targeted Analyses

To select SNPs for the targeted analyses, two meta‐analyses of influenza [4] and COVID‐19 [24] were considered. SNPs with a minor allele frequency (MAF) of > 0.2 (either directly genotyped or imputed) were included in the targeted analyses. This MAF threshold was selected based on power calculations described in section 3.7. In the influenza meta‐analysis [4], only rs12252 met the standard GWAS genome‐wide significance cut‐off of *p* < 5.0 × 10**^−^** ^8^. However, this SNP was not present in our dataset, and suitable proxies were not available (see supplementary methods for details). In the COVID‐19 meta‐analysis [24], 49 independent lead SNPs showed significant genome‐wide associations with critical COVID‐19. Twenty‐one of these SNPs were present in our dataset (5 directly genotyped and 16 imputed). After applying a MAF cut‐off of 0.2 (described below), 10 SNPs remained eligible for association analysis in our dataset (Supporting Information: Table S1).

### 2.5 Multiple Testing Correction and Adjusted Level of Significance

We used Bonferroni's correction for multiple testing. The alpha (0.05) was split, so 0.01 (corrected for the number of SNPs included in the targeted analyses i.e., *p* < 1 × 10**^−^** ^3^) was used as the significance cut‐off for the targeted analyses and 0.04 (corrected for multiple testing i.e., *p* < 4 × 10**^−^** ^8^) was used for the GWAS.

### 2.6 Subgroup Sensitivity Analysis

Sensitivity analyses included subgroup analyses of (1) individuals with PCR‐confirmed influenza (hospitalized cohort only), and (2) individuals < 65 years of age. The age threshold of 65 was selected as individuals at or above this age are at a higher risk of severe influenza‐related outcomes or complications [28], and therefore, the < 65 age group analysis may identify associations more clearly that are not confounded by the age‐driven effects. For the GWAS, a sensitivity analysis was also performed on the subgroup of individuals with European genetic ancestry, which was the only major ancestry group with sufficient outcome events. Genetic ancestry groups are defined as described in the supplementary methods section “population‐specific heterozygosity rate outliers.”

### 2.7 Calculations of Statistical Power for Targeted Analyses

To achieve reasonable statistical power for the detection of potential human SNP associations, we restricted the inclusion of SNPs in the targeted analyses based on power calculations conducted a priori with the genpwr package (1.0.4) using R software (4.2.0) [29]. Thus, we applied a MAF cut‐off criteria in the selection of SNPs for targeted analyses of at least 0.2, which gave us ~80% power to detect an odds ratio of approximately 1.8 in the hospitalized cohort. For the pooled cohort, this increased to 94%. We also assessed the power for associations performed only in the outpatients FLU 002 Plus study, and determined the power was too low for further assessment of this cohort independently.

### 2.8 Calculations of Statistical Power for GWAS

For the GWAS in the hospitalized cohort, power calculations indicated ~9.5% power to detect an odds ratio of 1.8 with a MAF of at least 0.2. To detect a lower MAF of 0.05 with a constant odds ratio of 1.8, the power decreased to 0.1%. To detect a lower odds ratio of 1.2 with the constant MAF of 0.2, the power decreased to 0.001%. For GWAS in the pooled cohort, power calculations indicated 26% power to detect an odds ratio of at least 1.8 with MAF of 0.2. For MAF of 0.05 and odds ratio of 1.8, power was 0.36% and for MAF of 0.2 and odds ratio of 1.2, power was 0.007%.

This study follows the strengthening the reporting of observational studies in epidemiology (STROBE) guidelines.

## 3 Results

### 3.1 Study Population

The hospitalized cohort consisted of 1634 individuals recruited into the FLU 003 Plus study across five continents (Table 1). The majority were white (54.6%), with 31.9% of individuals enrolled in Europe and 22% enrolled in Asia. The median age at enrollment was 59 years, with 62.3% aged under 65 years. Additionally, 71.8% of individuals had at least one chronic co‐morbidity, and 84.9% had PCR‐confirmed influenza by central or local laboratory determination.

**Table 1:** Baseline characteristics.

	Hospitalized cohort	Pooled cohort
Total persons (*n*)	1634	3469
FLU002 outpatients *n* (%)	n/a	1835 (52.9)
FLU003 hospitalized *n* (%)	1634 (100)	1634 (47.1)
Confirmed Influenza via PCR *n* (%)^a^	1388 (84.9)	3022 (87)
Time of enrollment, *n* (%)		
Enrollment in 2012–2013	140 (8.6)	463 (13.3)
Enrollment in 2014–2015	619 (37.9)	1484 (42.8)
Enrollment in 2016–2017	520 (31.8)	1167 (33.6)
Enrollment in 2018–2020	355 (21.7)	355 (10.2)
Age, median (IQR), years	59 (44, 72)	48 (35, 62)
18–44 years	411 (25.2)	1505 (43.4)
45–54 years	263 (16.1)	637 (18.4)
55–64 years	344 (21.1)	596 (17.2)
65–74 years	296 (18.1)	382 (11.0)
≥ 75 years	320 (19.6)	349 (10.1)
Sex (female)	868 (53.1)	1804 (52.0)
BMI, median (IQR), kg/m^2^	25.7 (22.0, 30.5)	25.3 (22.1, 29.4)
Obesity (BMI > 30), *n* (%)	447 (27.4)	747 (22.9)
Smoking status, *n* (%)		
Never smoked	845 (51.7)	2021 (58.3)
Former smoker	477 (29.2)	793 (22.9)
Active smoker before hospitalization	278 (17.0)	599 (17.3)
Pregnancy (proportion of women)	41 (4.7)	61(3.4)
Pregnant in third trimester (proportion of women)	20 (2.3)	24 (1.3)
Comorbidities, *n* (%)		
Patients with at least one comorbidity	1173 (71.8)	1726 (49.8)
Asthma	312 (19.1)	390 (11.2)
COPD^b^	345 (21.1)	370 (10. 7)
Diabetes	335 (20.5)	410 (11.8)
Cardiovascular disease (other than hypertension)	315 (19.3)	359 (10.3)
Chronic renal disease	163 (10.0)	171 (4.9)
Chronic liver disease	64 (3.9)	78 (2.2)
HIV	92 (5.6)	315 (9.1)
Other immunosuppressive condition or treatment	123 (7.5)	132 (3.8)
Race, *n* (%)		
White	892 (54.6)	1983 (57.2)
Asian	438 (26.8)	961 (27.7)
Black	142 (8.7)	241 (6.9)
Hispanic	100 (6.1)	211 (6.1)
Other	62 (3.8)	73 (2.1)
Continent of study enrollment, *n* (%)		
Europe	522 (31.9)	1345 (38.8)
North America	357 (21.8)	531 (15.3)
South America	91 (5.6)	415 (12.0)
Asia	359 (22.0)	857 (24.7)
Australia	305 (18.7)	321 (9.3)
Patients in ICU/on MV at study enrollment, *n* (%)	99 (6.1)	99 (2.8)

Abbreviations: COPD, chronic obstructive pulmonary disease; ICU, intensive care unit; MV, mechanical ventilation; N/A, not applicable.

^a^ Total PCR positives, including local and central determination.

^b^ Includes patients with “other chronic lung disease.”

Of the 4086 participants eligible for the pooled analysis from the FLU 002 Plus (outpatients) cohort, only 45% (*n* = 1835) had PCR‐confirmed influenza by central or local laboratory determination; the remaining 55% (*n* = 2251) were influenza negative and as the FLU 002 Plus did not require a clinician validated diagnosis of influenza (unlike FLU 003) these individuals were excluded from further analysis. Thus, the pooled cohort consisted of 3469 individuals: 52.9% (*n* = 1835) from FLU 002 Plus (outpatients) and 47.1% (*n* = 1634) from FLU 003 Plus (hospitalized). The majority were white (57.2%), with 38.8% enrolled in Europe. The median age at enrollment was 48 years, with 78.9% under 65 years of age. Additionally, 49.8% had at least one co‐morbidity.

In the hospitalized cohort, 148 (9.1%) individuals experienced disease progression by day 28. Of these, 87 individuals had a prolonged hospitalization, and 34 died (Table 2). In the pooled cohort, 189 (5.4%) individuals experienced disease progression, and 34 died (Table 2).

**Table 2:** Primary composite outcome of disease progression.

	Hospitalized cohort	Pooled cohort
Total patients (*n*)	1634	3469
Disease progression (*n*)	148a,b	148
Prolonged hospitalization (*n*)	87	87
Progression to MV/ICU (patients enrolled in general care unit) (*n*)	35	35
Death (*n*)	34	34
Time to death, mean (range), days	9.8 (1–27)	9.8 (1–27)
Hospitalization (*n*)	n/a	41
Total events (*n*)	148	189

Abbreviations: ICU, intensive care unit; MV, mechanical ventilation; N/A, not applicable.

^a^ Five patients in ICU died.

^b^ Three patients were in both individual outcomes of “Prolonged hospitalization” and “Progression to MV/ICU.”

### 3.2 Nongenetic Risk Factors Associated With Disease Progression

Considering nongenetic risk factors among the hospitalized cohort, older individuals (OR: 1.20 per 10‐year increase, 95%CI: 1.08–1.33, *p* < 0.001) were more likely to experience disease progression. There was also variation across continents, with those enrolled in Asia having lower odds ratios for developing disease progression than in Europe (OR: 0.24, 95%CI: 0.13–0.45, *p* < 0.001) (Supporting Information: Table S2). However, there were no significant differences by self‐reported race (overall *p* = 0.62; see pairwise comparisons in Supporting Information: Table S3).

Additionally, those with cardiovascular disease other than hypertension (OR: 1.43, 95%CI: 0.94–2.17, *p* = 0.098), chronic renal disease (OR: 1.54, 95%CI: 0.94–2.54, *p* = 0.088), and chronic liver disease (OR: 1.83, 95%CI: 0.90–3.73, *p* = 0.096) were associated with an increased risk of disease progression in the hospitalized cohort (Supporting Information: Table S3), although they did not reach statistical significance (*p* < 0.05). Thus, we did not adjust for any additional factors in the targeted analyses or GWAS in addition to those decided a priori (age, sex, and principal components 1–5 representing genetic ancestry).

### 3.3 Targeted Analyses

None of the 10 SNPs assessed in the targeted analysis were significantly associated with disease progression in either the hospitalized or the pooled cohort (Table 3). The results were consistent in sensitivity analyses of the subset of hospitalized patients with PCR‐confirmed influenza as well as those < 65 years of age (Supporting Information: Table S4). In the analysis of the hospitalized cohort, rs2071590 had the highest odds ratio: 1.24 (95%CI: 0.97–1.58; *p* = 0.09), which increased in sensitivity analysis of individuals with PCR‐confirmed influenza: 1.28 (95%CI: 0.97–1.68; *p* = 0.08), and individuals < 65 years of age: 1.46 (95%CI: 1.04–2.03; *p* = 0.03). Similarly, in the pooled cohort, the odds ratio for rs2071590 was 1.29 (95%CI: 1.04–1.61; *p* = 0.02) and 1.47 in individuals < 65 years of age (95%CI: 1.11–1.95, *p* = 0.01, Supporting Information: Table S5). In addition to rs2071590, two more SNPs in the pooled cohort sensitivity analysis of individuals < 65 years of age had 95% confidence intervals that did not cross 1; rs1073165 (1.41, 95%CI: 1.06–1.88, *p* = 0.02) and rs879055593 (1.48, 95%CI: 1.08–2.02, *p* = 0.01) (Supporting Information: Table S5).

**Table 3:** Targeted SNP analysis.

	Hospitalized cohort						Pooled cohort					
**SNP ID (rsID)**	**Observed count**	**MAF**	**OR**	**Lower 95% CI**	**Upper 95% CI**	***p* value**	**Observed count**	**MAF**	**OR**	**Lower 95% CI**	**Upper 95% CI**	***p* value**
rs11706494	1607	0.28	1.03	0.79	1.35	0.81	3377	0.28	1.03	0.82	1.31	0.78
rs1073165	1618	0.34	1.01	0.78	1.30	0.96	3402	0.33	1.11	0.89	1.38	0.36
rs2071590	1631	0.30	1.24	0.97	1.58	0.09	3423	0.31	1.29	1.04	1.61	0.02
rs2897075	1605	0.36	0.99	0.76	1.28	0.92	3357	0.37	0.94	0.75	1.19	0.62
rs879055593	1629	0.21	1.17	0.88	1.56	0.28	3419	0.21	1.22	0.95	1.55	0.12
rs721917	1631	0.48	1.13	0.89	1.45	0.32	3423	0.47	1.07	0.86	1.32	0.55
rs61882275	1552	0.38	1.10	0.86	1.41	0.45	3263	0.39	1.07	0.86	1.33	0.54
rs2660	1631	0.29	1.06	0.81	1.38	0.67	3423	0.29	1.13	0.90	1.42	0.31
rs12941811	1588	0.38	0.92	0.72	1.19	0.55	3347	0.40	0.91	0.73	1.14	0.42
rs35463555	1617	0.28	0.80	0.60	1.05	0.11	3401	0.29	0.82	0.64	1.05	0.11

*Note:* The adjusted alpha (significance level) for multiple comparison in our targeted analysis is 0.001.

Abbreviations: CI, confidence interval; MAF, minor allele frequency; OR, odds ratio.

### 3.4 GWAS

**Figure 1:**
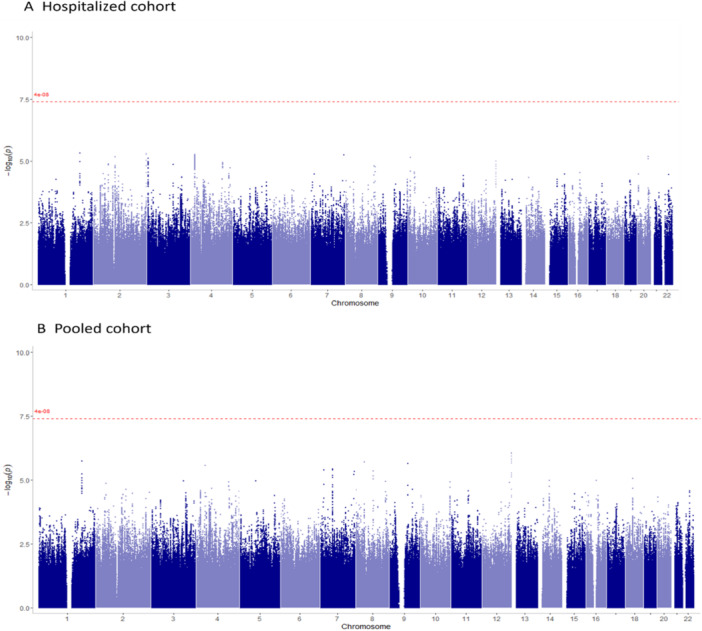
Manhattan plot from the analysis of the Hospitalized (A; *n* = 148 cases and 1483 controls) and Pooled (B; *n* = 188 cases and 3235 controls) cohorts. The Manhattan plot shows the individual SNPs, organized by chromosome on the *x*‐axis, and the negative log *p*‐values on the *y*‐axis. The figure colors alternate by chromosome for visualization of the chromosome boundaries. The dotted red line represents the genome‐wide significance cut‐off used in this study (*p* < 4 × 10**^−^** ^8^). No SNP reached the genome‐wide significance cut‐off in either of the two cohorts assessed.

The results of GWAS for both the hospitalized and pooled cohorts are shown in Manhattan plots (Figure 1, QQ plots are in Supporting Information: Figure S1). As in the targeted analyses, no SNPs were significantly associated with disease progression in GWAS of either the hospitalized or pooled cohorts. QQ and Manhattan plots for the sensitivity analyses are shown in Supporting Information: Figures S1 and S2, respectively. As in the main analysis, no SNPs passed our threshold for statistical significance but in the pooled cohort, sensitivity analysis for individuals < 65 years of age, two peaks, in intergenic regions of chromosome 14 and 7, approached the predefined genome wide significance threshold, rs2481897 (*p* = 1.13 × 10**^−^** ^7^) and rs62453029 (*p* = 1.84 × 10**^−^** ^7^), respectively. Likewise, the sensitivity analysis including only those of European decent showed no significant results.

## 4 Discussion

We applied targeted analyses and a GWAS using a geographically diverse, multi‐ethnic cohort of individuals with suspected or laboratory‐confirmed influenza to assess human SNP associations with disease progression. In the targeted analyses of 10 SNPs with previous evidence of impact on critical COVID‐19, we did not detect any significant association with disease progression in either the hospitalized or pooled cohort. However, some associations were observed in sensitivity analyses and were strongest in the subgroup of individuals aged < 65 years, but did not reach our significance threshold of *p* < 0.001. In the GWAS, no SNP associations with disease progression were identified in either cohort or in the sensitivity analyses. Our results are in line with the inconsistent results of prior genetic association studies into host genetic contribution to risk of severe influenza.

Statistical power to detect SNP associations was a central consideration for the study design. This led to the decision to perform targeted analyses in addition to a GWAS. To be able to perform both types of analyses within the same study, we took the cautious approach of splitting the alpha between the targeted analyses and the GWAS and controlled for multiple testing using the Bonferroni correction for each analysis. This allowed an assessment of *a priori* identified SNPs with plausible biological association with the studied phenotype using a less conservative cut‐off than traditional GWASs, while also allowing for the exploration of all other SNPs with a control for type 1 error consistent with the number of assessments performed. As detailed in the methods section, we had reasonable statistical power to detect an association for one or more of the 10 SNPs included in the targeted analyses, whereas the statistical power to detect SNPs in the GWAS was limited. Reflecting on our sample size and rate of disease progression, it may be that true associations of SNPs with disease progression have low effect sizes and/or with MAFs, and this study was underpowered to detect these associations. We also opted to conduct a secondary analysis in a pooled cohort of outpatients and hospitalized individuals, giving us a larger sample size but a less strictly defined phenotype. However, the pooled cohort analysis was in line with previous work analyzing the FLU 002 Plus and FLU 003 Plus cohorts [27] and reflects an underlying hypothesis that disease progression in both hospitalized and outpatients have similar biological mechanisms.

In the targeted analyses, we aimed to take advantage of the knowledge gained in large international collaborations investigating individuals with critical COVID‐19 [24]. However, SNPs associated with severe COVID‐19 may reflect a different host immune response involved in SARS‐CoV‐2 infection from the immunogenetic pathways underlying disease progression due to Influenza. This may be because the primary infection by SARS‐CoV‐2 impacts disease progression more strongly than in Influenza and other vURTIs where comorbidities, age, and secondary infections play a greater role. Hence, the impact of host SNPs relating to antiviral response may be diluted in a non‐COVID vURTI cohort. From our results, the sensitivity analyses in individuals aged < 65 years may lend support to this hypothesis, although we still did not observe any significant associations at our predefined threshold, the sensitivity analysis showed larger effect sizes for some of the targeted SNPs. Additionally, in the pooled cohort, we observed two peaks that approached genome‐wide significance. These regions and target SNPs may be useful starting points for future studies to identify genetic correlates of disease progression, which should also carefully consider the heterogeneity of the cohort to be studied, particularly with regards to age.

One of the SNPs included in targeted analyses, rs2071590 (A > G) showed a marginal, but nonstatistically significant signal (OR: 1.24, 95%CI: 0.97–1.58; *p* = 0.09), which increased in sensitivity analysis of individuals < 65 years of age: 1.46 (95%CI: 1.04–2.03; *p* = 0.03). Interestingly, the magnitude of effect size and subsequent increase when restricted to those aged < 65 was similar for both the hospitalized and the pooled cohort, suggesting that there may be common mechanisms for disease progression in hospitalized and outpatients, although it's important to note that the pooled cohort contained many of the same patients as the hospitalized cohort. rs2071590 is an intron variant in the promotor region of the lymphotoxin alpha (*LTA*) gene (also known as tumor necrosis factor [TNF‐beta]) on chromosome 6 (Gene ID: 4049) [30] in the major histocompatibility complex (MHC) class III region. The *LTA* protein is a cytokine produced by lymphocytes and a member of the TNF superfamily, which is involved in inflammatory, immunostimulatory, as well as antiviral responses [31]. In the critical COVID‐19 meta‐analysis [24], the rs2071590 (A > G) association showed an OR of 1.1 (95%CI: 1.06–1.11, *p* = 3.1 × 10^−10^). In addition to the association of LTA with critical COVID‐19, prior genetic association studies in a Mexican population have shown rs909253 (A > G) in *LTA* (as well as in SNPs in the related cytokine, *TNF*) associated with increased susceptibility to severe influenza A/H1N1, as well as a borderline significant association with mortality [31, 32]. The genetic variation in the MHC class III region, including *LTA* and *TNF*, has important impact in the immune response as well as inflammation and antigen presentation, and thus, genetic variation in the MHC III region may affect the progression of both RNA virus infections: COVID‐19 and influenza. In the sensitivity analysis of the pooled cohort, two additional SNPs had confidence intervals that did not cross 1 in the targeted analysis: rs879055593 in the ABO gene and the intergenic SNP rs1073165. Further, two peaks approached genome‐wide significance in the GWAS study, rs2481897 and rs62453029 (both intergenic). However, given that none of these SNPs passed the significance threshold used in this study, and all but rs2071590 were only of interest in sensitivity analyses, these results should be interpreted with caution until larger studies can shed more light on the impact of these SNPs on diseases progression in persons with influenza.

The strengths of the study included the large sample size of individuals, in comparison to prior genetic association studies in influenza, from five continents: Europe, North and South America, Asia, and Australia, and well‐defined outcome with reliable ascertainment via case report forms. Conversely, there were several limitations in the study. The association analyses were challenged by the diversity of study participants with respect to age, co‐morbidities, Influenza season, and the development of secondary conditions with potential effect on the outcome of disease progression. In addition, we observed clear difference in the rates of disease progression among individuals enrolled from different continents, which may be due to differences in hospitalization protocols (based on the number of available hospital beds, medical practice, etc.). Further, the phenotype of individuals developing disease progression included both death, progression to ICU/MV, and prolonged hospitalization, but was largely driven by prolonged hospitalization, which may have been due to factors other than the influenza. Due to a low event rate of disease progression of 9% (*n* = 148) and 5% (*n* = 189) in the hospitalized and pooled cohort, respectively, the statistical power of the targeted analyses, as well as the GWAS was limited. The cohort sample size restricted our power to analyse genotypes with odds ratios below 1.8. The 10 SNPs associated with severe COVID‐19 included in the targeted analyses showed odds ratios as low as 1.1 in the COVID‐19 meta‐analysis [24]. Therefore, if the true effect size was similar for influenza, we would have had insufficient power to detect these associations. Further, in the targeted analysis, a number of SNPs were not present in our imputed genetic data, including rs12252, which has been previously reported to be associated with severe influenza [4], and 28 of the 49 SNPs significantly associated with critical Covid‐19 [24]. To obtain larger numbers of individuals developing severe progression of influenza infection, collaborations across cohort studies should be considered. This has shown to be of benefit in the large international COVID‐19 studies, for example, The COVID‐19 host genetics initiative [33]. Complementary to this, alternative approaches to selecting SNPs for targeted analyses, such as a recent multi‐omic approaches performed using publicly available transcriptomics data and a combination of proteomics and siRNA knockdowns of human cell lines [34], should also be considered moving forward [35, 36]. These studies identified putative host genes involved in influenza pathogenesis, including two genes, CDK2 and FLT3, that show effect across influenza and coronavirus families. Cross‐validation of these and future similar approaches in large collaborative host genetic cohorts would be valuable. A final limitation of this study is that for any GWAS built upon SNP‐array genotype data, the ability to detect rare variants was limited.

## 5 Conclusion

In a large, multi‐ethnic cohort of individuals with known or suspected influenza, we were unable to detect associations between SNPs and disease progression. In line with the conflicting outcomes of prior genetic association studies into vURTIs, any single SNP associated with disease progression may have a smaller effect size (and a corresponding lower MAF), which the present study was underpowered to detect. Multi‐cohort collaborations and meta‐analyses to increase statistical power, as has been done extensively in COVID‐19 host genetic research, are therefore crucial to progress this field.

## Author Contributions

**Sara Bohnstedt Mørup:** writing – review and editing, writing – original draft, methodology, data curation. **Maja Milojevic:** writing – review and editing, visualization, methodology, formal analysis. **Seyed Mahmood Taghavi Shahri:** writing – review and editing, formal analysis, data curation. **Brad T. Sherman:** methodology, writing – review and editing, resources. **Weizhong Chang:** methodology, writing – review and editing, resources. **Ruth Lynfield:** methodology, writing – review and editing, resources. **Marie Helleberg:** methodology, writing – review and editing, resources. **Lars Østergaard:** methodology, writing – review and editing, resources. **Line Borgwardt:** methodology, writing – review and editing, supervision. **Norman Gerry:** methodology, writing – review and editing, resources. **Marcelo Losso:** methodology; writing – review and editing, resources. **Kenneth Baillie:** methodology, writing – review and editing, resources. **Richard Davey:** methodology, writing – review and editing, resources. **Dominic E. Dwyer:** methodology, writing – review and editing, resources. **Chansavath Phetsouphanh:** methodology, writing – review and editing, resources. **Vasileios Papastamopoulos:** methodology, writing – review and editing, resources. **Richard M. Novak:** methodology, writing – review and editing, resources. **Daniel D. Murray:** conceptualization, writing – review and editing, methodology, supervision, resources. **Joanne Reekie:** conceptualization, writing – review and editing, methodology, resources, supervision. **Jens D. Lundgren:** conceptualization, writing – review and editing, methodology, resources, supervision. **H. Clifford Lane:** conceptualization, methodology, writing – review and editing, resources. **Cavan Reilly:** conceptualization, writing – review and editing, methodology, resources.

## Ethics Statement

Written consent to participate in research and the comprehensive genetic analysis was obtained from all individuals before study inclusion. The participating sites received approvals from the Institutional Review Board (IRB) or Ethics Committee (EC) before their implementation. All study individuals' confidentiality will be protected in accordance with standard IRB/EC policies and procedures.

## Conflicts of Interest

The authors declare no conflicts of interest.

## Supporting information


**Supplemental Figure 1:** Quantile‐Quantile plots of GWAS results for Hospitalized. **Supplemental Figure 2:** Manhattan plots from the sensitivity analysis of the Hospitalized cohort for participants with PCR confirmed influenza. **Supplemental Table 1:** Targeted SNPs. **Supplemental Table 2:** The main non‐genetics prediction model using Baseline Age (unit: 10‐years), Sex (ref: Female), and Continent* (reference: Europe). **Supplemental Table 3:** The adjusted effects of non‐genetic factors which were estimated by adding each variable to the main non‐genetics model separately*. **Supplemental Table 4:** Sensitivity analysis for the targeted SNPs in the hospitalized cohort. **Supplemental Table 5:** Sensitivity analysis for the targeted SNPs in the pooled cohort for individuals under age 65 (*n* = 105 cases and 2594 controls).

## Data Availability

Data from this study can be made available by contacting the STRIVE Scientific Steering Committee via the following form https://www.insight-trials.org/research_proposal/strive/.
