# Supplementary material for: Investigating the Impact of Host Genetics on the Risk of Disease Progression in Individuals With Influenza
Source: Immun Inflamm Dis. 2026 Mar 11;14(3):e70394. doi: 10.1002/iid3.70394 (PMC13098070; doi:10.1002/iid3.70394)
Supplement: Supplementary file 1 — Supplemental Figure 1: Quantile‐Quantile plots of GWAS results for Hospitalized. Supplemental Figure 2: Manhattan plots from the sensitivity analysis of the Hospitalized cohort for participants with PCR confirmed influenza. Supplemental Table 1: Targeted SNPs. Supplemental Table 2: The main non‐genetics prediction model using Baseline Age (unit: 10‐years), Sex (ref: Female), and Continent* (reference: Europe). Supplemental Table 3: The adjusted effects of non‐genetic factors which were estimated by adding each variable to the main non‐genetics model separately*. Supplemental Table 4: Sensitivity analysis for the targeted SNPs in the hospitalized cohort. Supplemental Table 5: Sensitivity analysis for the targeted SNPs in the pooled cohort for individuals under age 65 (n = 105 cases and 2594 controls). [file IID3-14-e70394-s001.docx]

**SUPPLEMENTARY**

Study cohort genotype data and QC

Human DNA from participants´ blood samples were genotyped using a custom Affymetrix array (including 770,558 probes), which was enriched with SNPs in genes related to immune dysfunction. Genotyping and subsequent quality assurance was performed by Advanced Biomedical Laboratories (ABML).

Quality control (QC) of genetic data

In total, 5771 unique *INSIGHT* Genomics Study samples were genotyped and met the QC inclusion criteria of a quality control call rate ≥97%, an autosomal call rate ≥97%, and agreement between reported and genetically determined sex. *INSIGHT* Genomics Study samples included participants within the FLU 003+ cohort as well as participants in the outpatient study, FLU 002+. After filtering SNP probes to exclude duplicate or multiallelic loci, and probes that were not recommended for use by Affymetrix, 670,364 autosomal markers remained for use in SNP imputation. Cryptic relatives were identified using KING kinship coefficients.

SNP Imputation and post imputation QC

SNP imputation to the 1000 Genomes phase 3[1] (genome build: GRCh37/hg19) was performed on quality-controlled genotype data with the genipe pipeline using PLINK (v2.00a3LM)[2] and SHAPEIT (v2.5)[3] for phasing and IMPUTE2 (v2.3.2)[4] for imputation. Imputed SNPs with a confidence score INFO ≤0.8 and duplicates were removed. SNPs fulfilling any of the following were excluded: (a) MAF<5%, (b) genotype missing rate >5%, or (c) Hardy–Weinberg equilibrium p value <1x10^−6^. Samples with >10% missing SNPs were also excluded. 3,556,582 variants remained.

Principal components and ADMIXTURE

The first 10 principal components for the FLU cohort and 1000 Genomes reference populations were calculated using PLINK (v1.9 and v2.0) on non-ambiguous, biallelic, directly genotyped autosomal SNPs with a MAF ≥1% and a genotyping rate ≥90%. High LD region removal and pairwise-pruning (window size of 50 variants, step size of 5 variants, and r^2^ threshold of 0.2) were performed prior to generating the PCs. ADMIXTURE[4] was run on the same input SNPs as for the PCA, with K ancestral populations set to five. ADMIXTURE values for each K population were overlayed on the first two principal components to confirm that the ADMIXTURE-defined populations were in alignment with the five 1000 Genomes superpopulations.

Population-specific heterozygosity rate outliers

Samples with an ADMIXTURE fraction of at least 0.7 attributable to a 1000 Genomes superpopulation (i.e. European, East Asian, South Asian, African, Admixed American) were assigned to that population and were considered outliers if they had a heterozygosity rate greater than 3 SD from the population-specific mean. The samples which were not assigned to a single superpopulation were considered ‘Admixed’ and were marked as outliers if they had a heterozygosity rate exceeding 3 SD from the whole cohort mean. Select excluded samples (n=8) were reintroduced to the dataset after visual inspection of a 3D plot of PC1, PC2, and the heterozygosity rate indicated that the samples were likely assigned as outliers due to population attribution cut-offs rather than poor sample quality. Fifty samples were removed as heterozygosity rate outliers, leaving a total of 5721 samples.

Performing the GWAS

The GWAS was performed on the post imputation QC’ed SNPs using the --glm option in PLINK 2.0[2]. The first 5 principal component covariates were transformed to have a mean of zero and variance of 1 with the --covar-variance-standardize option. Cryptic relatedness was accounted for by excluding one individual from each pair of first-degree relatives (kinship coefficient ≥0.177; n=2 pairs). The individual SNP variables were assessed using the additive model, i.e., as a continuous variable (0, 1 or 2), and the significance of the associations were assessed. For the interpretation of SNP associations´ significance, we used a predefined adjusted level of significance by splitting the alpha between the targeted analyses and most SNPs included in the GWAS (described below). 95% confidence intervals were calculated with PLINK’s --ci option. Quantile-Quantile (Q-Q) plots and Manhattan plots were generated with the qqman[5] and topr[6] packages in R software[7] (version 4.2.0).

SNPs for inclusion in targeted analyses

A number of SNPs were not present in our dataset, including rs12252 in *IFITM3* (the only SNP previously significantly associated with severe influenza) and 28 of 49 SNPs significantly associated with critical Covid-19 in meta-analysis[8]. For the purpose of SNPs to be used for proxies, we investigated linkage disequilibrium (LD) of *IFITM3* SNPs, including rs12252 and rs34481144[9]. We did not detect any SNPs which were in high LD (defined as having an R^2^ of at least 0.8) with either of the two aforementioned *IFITM3* SNPs.

Supplemental Table 1 Targeted SNPs

| **SNP ID (rsID)** | **Chromosome and position (Hg38)** | **Position (Hg37)** | **Ref** | **Alt** | **Closest Gene (from** [10]**)** |
| --- | --- | --- | --- | --- | --- |
| **rs11706494** | 3:101790631 | 101509475 | A | T | *NXPE3* |
| **rs1073165** | 4:167824478 | 168745629 | A | G | − |
| **rs2071590** | 6:31571991 | 31539768 | G | A | *LTA* |
| **rs2897075** | 7:100032719 | 99630342 | C | T | *ZKSCAN1* |
| **rs879055593** | 9:133271182 | 136146597 | C | T | *ABO* |
| **rs721917** | 10:79946568 | 81706324 | A | G | *SFTPD* |
| **rs61882275** | 11:34482745 | 34504292 | G | A | *ELF5* |
| **rs2660** | 12:112919637 | 113357442 | A | G | *OAS1* |
| **rs12941811** | 17:40003082 | 38159335 | C | T | *PSMD3* |
| **rs35463555** | 19:50374423 | 50877680 | G | A | *NR1H2* |

**Abbreviations** - Alt: Alternate allele, Ref: Reference allele

Supplemental Table 2 The main non-genetics prediction model using Baseline Age (unit: 10-years), Sex (ref: Female), and Continent* (reference: Europe).

| Main model predictors | Adjusted Odds Ratio | Lower 95%CI | Upper 95%CI | P-value |
| --- | --- | --- | --- | --- |
| Baseline Age (Unit: 10-years) | 1.20 | 1.08 | 1.33 | <0.001 |
| Sex (Male) | 1.40 | 0.99 | 1.98 | 0.057 |
| Continent  North America | 0.65 | 0.41 | 1.03 | 0.065 |
| South America | 1.45 | 0.80 | 2.63 | 0.223 |
| Asia | 0.24 | 0.13 | 0.45 | <0.001 |
| Australia | 0.59 | 0.36 | 0.97 | 0.036 |

*The overall p-value for the continent calculated using a Likelihood Ratio test is < 0.0001.

Supplemental Table 3 The adjusted effects of non-genetic factors which were estimated by adding each variable to the main non-genetics model separately*.

| Non-Genetics Factors | Adjusted Odds Ratio | Lower 95%CI | Upper 95%CI | P-value |
| --- | --- | --- | --- | --- |
| Smoking status  Former vs never† | 0.90 | 0.60 | 1.37 | 0.63 |
| Active vs never† | 0.90 | 0.55 | 1.49 | 0.69 |
| Unknown vs never† | 0.67 | 0.19 | 2.29 | 0.52 |
| BMI (continuous) | 0.98 | 0.95 | 1.01 | 0.12 |
| Race  Asian vs White† | 0.69 | 0.27 | 1.72 | 0.43 |
| Black vs White† | 0.59 | 0.26 | 1.31 | 0.19 |
| Hispanic vs White† | 0.92 | 0.44 | 1.94 | 0.83 |
| Others vs White† | 1.16 | 0.46 | 2.92 | 0.76 |
| FLU types  Flu-A vs negative† | 0.73 | 0.49 | 1.09 | 0.12 |
| Flu-B vs negative† | 0.71 | 0.38 | 1.34 | 0.29 |
| Unknown vs negative† | 1.28 | 0.50 | 3.32 | 0.61 |
| Comorbidity (having any) | 1.12 | 0.73 | 1.72 | 0.61 |
| Number of comorbidities | 1.13 | 0.98 | 1.31 | 0.10 |
| Asthma | 0.71 | 0.43 | 1.17 | 0.18 |
| COPD/other chronic lung disease | 1.11 | 0.74 | 1.65 | 0.62 |
| Diabetes | 0.80 | 0.51 | 1.25 | 0.32 |
| Cardiovascular disease: other than hypertension | 1.43 | 0.94 | 2.17 | 0.098 < 0.1 |
| Chronic renal disease | 1.54 | 0.94 | 2.54 | 0.088 < 0.1 |
| Chronic liver disease | 1.83 | 0.90 | 3.73 | 0.096 < 0.1 |
| HIV | 1.00 | 0.44 | 2.27 | 1.00 |
| Other immunosuppressive condition or treatment | 1.08 | 0.59 | 2.00 | 0.80 |
| Pregnancy[^ǂ^](https://en.wikipedia.org/wiki/%C7%82) | 1.48 | 0.40 | 5.46 | 0.56 |

***** The observed protective effects (OR<1) should be interpreted with caution as they might be due to over-recruiting (hospitalization) of mild cases from that high-risk group of participants in the study. Please note the estimating model was not adjusted for any influenzas’ severity index at the onset of hospitalization.

**†** The overall p-values, calculated using Likelihood Ratio tests, are 0.89 for smoking, 0.62 for race, and 0.33 for FLU types.

[**ǂ**](https://en.wikipedia.org/wiki/%C7%82) In analysis of pregnancy, we used a subset of data including just women 45 years old or younger, n(subset)=238, n(event)=15.

**Abbreviations** - COPD: Chronic obstructive pulmonary disease

Supplemental Table 4 Sensitivity analysis for the targeted SNPs in the hospitalized cohort

|  | **Confirmed influenza sensitivity analysis (n=1386)** | | | | | | **Under 65 years of age sensitivity analysis (n=1017)** | | | | | |
| --- | --- | --- | --- | --- | --- | --- | --- | --- | --- | --- | --- | --- |
| **SNP ID (rsID)** | **Observed count** | **MAF** | **OR** | **Lower 95% CI** | **Upper 95% CI** | **P-value** | **Observed count** | **MAF** | **OR** | **Lower 95% CI** | **Upper 95% CI** | **P-value** |
| **rs11706494** | 1367 | 0.29 | 0.94 | 0.70 | 1.27 | 0.71 | 1002 | 0.28 | 1.08 | 0.75 | 1.55 | 0.67 |
| **rs1073165** | 1376 | 0.35 | 0.99 | 0.75 | 1.31 | 0.95 | 1009 | 0.34 | 1.27 | 0.89 | 1.81 | 0.19 |
| **rs2071590** | 1386 | 0.31 | 1.28 | 0.97 | 1.68 | 0.08 | 1017 | 0.30 | 1.46 | 1.04 | 2.03 | 0.03 |
| **rs2897075** | 1368 | 0.36 | 0.90 | 0.68 | 1.20 | 0.48 | 999 | 0.36 | 1.12 | 0.78 | 1.61 | 0.55 |
| **rs879055593** | 1385 | 0.21 | 1.08 | 0.79 | 1.49 | 0.63 | 1016 | 0.21 | 1.43 | 0.96 | 2.12 | 0.08 |
| **rs721917** | 1386 | 0.48 | 1.17 | 0.89 | 1.54 | 0.26 | 1017 | 0.50 | 0.95 | 0.68 | 1.34 | 0.79 |
| **rs61882275** | 1318 | 0.38 | 1.02 | 0.77 | 1.35 | 0.87 | 967 | 0.37 | 1.35 | 0.94 | 1.93 | 0.11 |
| **rs2660** | 1386 | 0.29 | 1.02 | 0.76 | 1.37 | 0.89 | 1017 | 0.27 | 0.99 | 0.67 | 1.47 | 0.98 |
| **rs12941811** | 1349 | 0.39 | 0.83 | 0.63 | 1.11 | 0.22 | 983 | 0.38 | 1.01 | 0.71 | 1.45 | 0.94 |
| **rs35463555** | 1375 | 0.28 | 0.80 | 0.59 | 1.09 | 0.16 | 1008 | 0.28 | 0.90 | 0.61 | 1.31 | 0.58 |

**Abbreviations** - CI: Confidence interval, MAF: Minor allele frequency, OR: Odds ratio

Supplemental Table 5 Sensitivity analysis for the targeted SNPs in the pooled cohort for individuals under age 65 (n=105 cases and 2594 controls)

| **SNP ID (rsID)** | **Observed count** | **MAF** | **OR** | **Lower 95% CI** | **Upper 95% CI** | **P-value** |
| --- | --- | --- | --- | --- | --- | --- |
| **rs11706494** | 2663 | 0.2807 | 1.06 | 0.79 | 1.43 | 0.69 |
| **rs1073165** | 2683 | 0.3317 | 1.41 | 1.06 | 1.88 | 0.02 |
| **rs2071590** | 2699 | 0.3118 | 1.47 | 1.11 | 1.95 | 0.01 |
| **rs2897075** | 2645 | 0.3667 | 0.99 | 0.73 | 1.33 | 0.93 |
| **rs879055593** | 2697 | 0.2154 | 1.48 | 1.08 | 2.02 | 0.01 |
| **rs721917** | 2699 | 0.4785 | 0.94 | 0.71 | 1.25 | 0.69 |
| **rs61882275** | 2572 | 0.39 | 1.23 | 0.92 | 1.65 | 0.16 |
| **rs2660** | 2699 | 0.2857 | 1.15 | 0.85 | 1.56 | 0.37 |
| **rs12941811** | 2635 | 0.3989 | 0.98 | 0.73 | 1.31 | 0.87 |
| **rs35463555** | 2682 | 0.2931 | 0.91 | 0.67 | 1.24 | 0.55 |

**Abbreviations** - CI: Confidence interval, MAF: Minor allele frequency, OR: Odds ratio

**Supplemental figures**


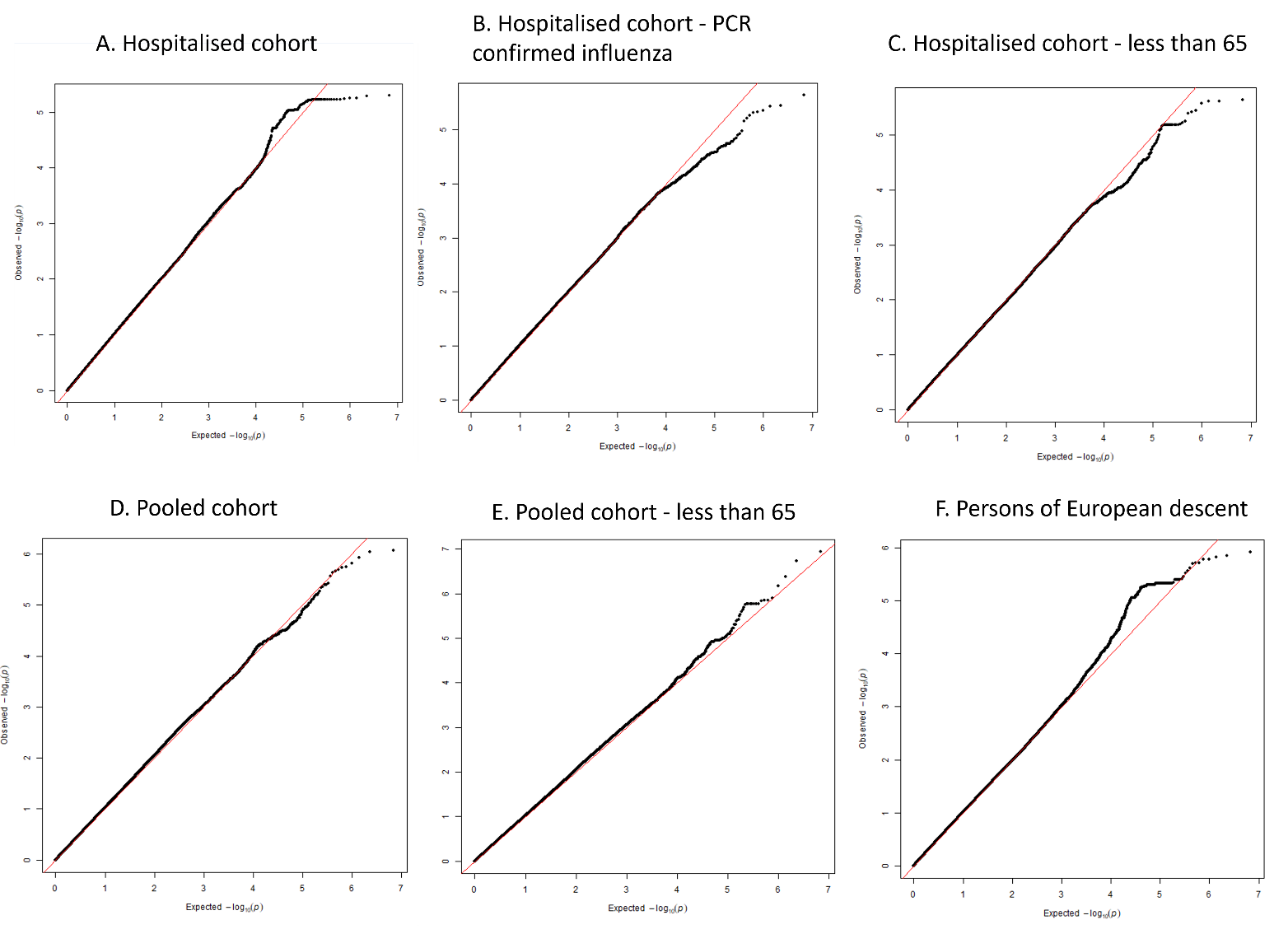
**Supplemental Figure 1 Quantile-Quantile plots of GWAS results for Hospitalised (A-C) and Pooled (D, E) cohorts.** Three GWASs were run for the Hospitalised cohort – A) The full Hospitalized cohort (n=148 cases and 1483 controls), B) Only PCR confirmed influenza cases (n= 120 cases and 1266 controls), C) only patients under age 65 (n=73 cases and 944 controls). For the Pooled cohort, a GWAS was run for the full cohort (D; n=188 cases and 3235 controls), for patients under age 65 (E; n=105 cases and 2594 controls), and persons of European descent (F; n=110 cases and 779 controls). Each point on the plot represents a SNP. The axes show the -log10 *p*-values from the observed and expected chi-square distributions. The red line represents where data points would be found under the null hypothesis of no association between a SNP and the outcome event. The genomic inflation factor (λ) for each GWAS as estimated based on the median chi-square statistic was close to 1, suggesting a lack of significant unaccounted for bias in the data due to reasons such as population stratification. The λ for each GWAS is as follows: A) λ=1.02, B) λ=1.01, C) λ=1.02, D) λ=1.03, E) 1.04, F) λ=1.01.

**
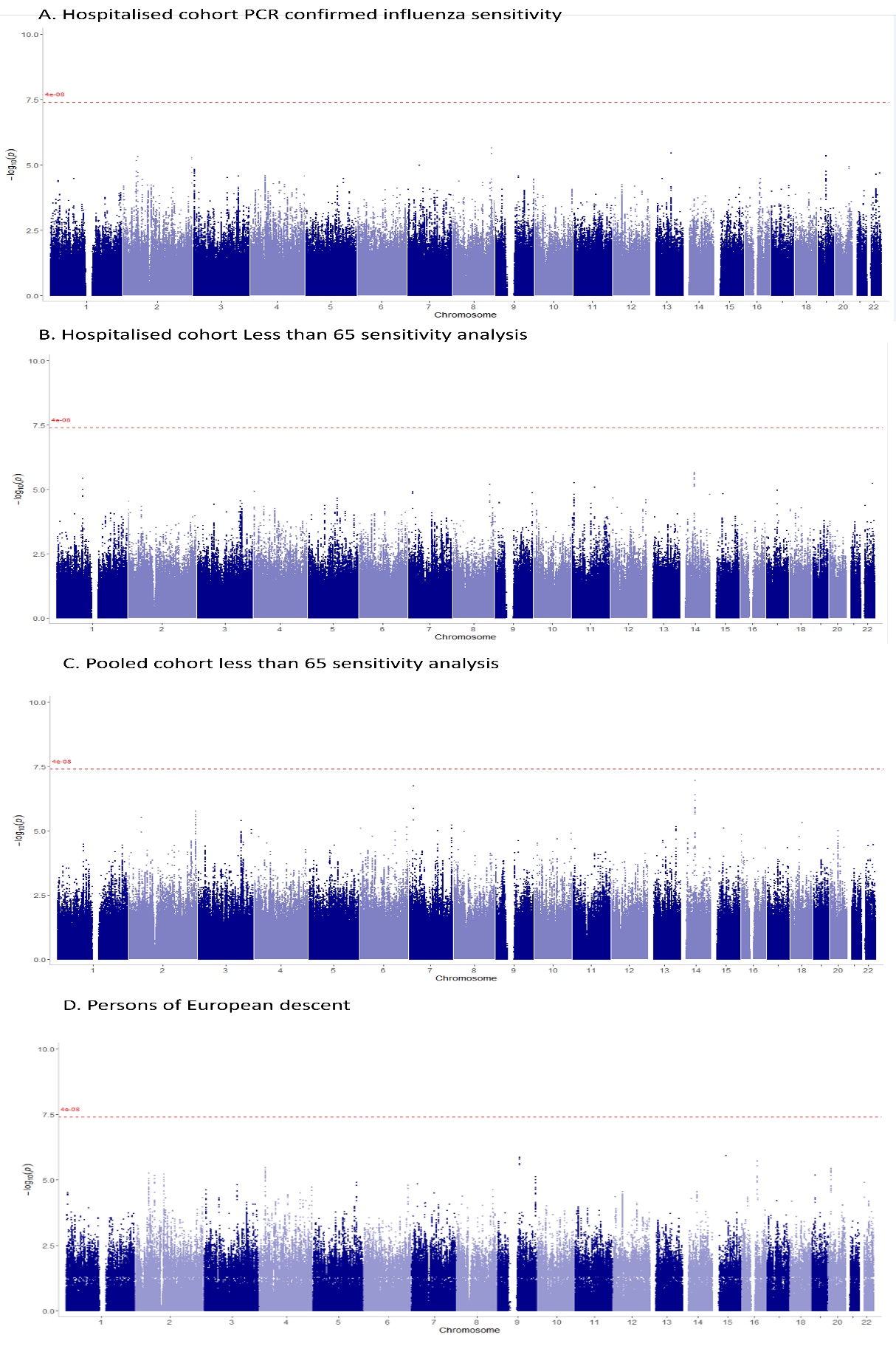
**

**Supplemental Figure 2 Manhattan plots from the sensitivity analysis of the Hospitalised cohort for participants with PCR confirmed influenza (A; n=120 cases and 1266 controls) and participants under age 65 (B; 73 cases and 944 controls), participants under age 65 from the Pooled cohort (C; n=105 cases and 2594 controls), and persons of European descent (D; n=110 cases and 779 controls).** The Manhattan plot shows the individual SNPs, organized by chromosome on the x axis, and -log p-values on the y axis. The figure colours alternate by chromosome for visualization of the chromosome boundaries. The dotted redline represents the genome wide significance cut-off used in this study (p < 4 x 10^-8^). No SNP reached the genome-wide significance cut off in the four sensitivity analyses.

The INSIGHT FLU002 and FLU003 Study Group

International coordinating centers:

Copenhagen: Bitten Aagaard, Álvaro H. D. Borges, Alessandro Cozzi-Lepri, Marius Eid, Per O. Jansson, Marianne Jeppesen, Zillah Maria Joensen, Ruth Kjærgaard Pedersen, Jens Lundgren, Birgit Riis Nielsen, Mary Pearson, Lars Peters, Tavs Qvist.

London: Brian Angus, Abdel Babiker, Rachel Bennett, Nafisah Braimah, Yolanda Collaco-Moraes, Adam Cursley, Fleur Hudson, Sarah Pett, Charlotte Russell, Helen Webb.

Sydney: Dianne Carey, David Courtney-Rodgers, Sean Emery, Pamela Shaw.

Washington: Fred Gordin, Adriana Sanchez, Barbara Standridge, Michael Vjecha.

Statistical and Data Management Center, Minneapolis, Minnesota: Kate Brekke, Megan Campbell, Eileen Denning, Alain DuChene, Nicole Engen, Michelle George, Merrie Harrison, James D. Neaton, Ray Nelson, Siu-Fun Quan, Terri Schultz, Deborah Wentworth.

Specimen repositories and laboratories: John Baxter, Shawn Brown (Leidos Biomedical Research, Inc.), Marie Hoover (ABML).

National Institute of Allergy and Infectious Disease/Leidos: John Beigel, Richard T. Davey Jr., Robin Dewar, Erin Gover, Rose McConnell, Julia Metcalf, Ven Natarajan, Tauseef Rehman, Jocelyn Voell.

Institute of Clinical Pathology and Medical Research, NSW Health Pathology, Westmead Hospital and University of Sydney, Westmead, New South Wales, Australia: Dominic E. Dwyer, Jen Kok.

Centers for Disease Control and Prevention, Atlanta, Georgia: Timothy M. Uyeki.

Community representative: David Munroe.

Clinical site investigators by country (n = number of participants enrolled):

Argentina (n = 1567): Damian Aguila, Maria Fernanda Alzogaray, Maria Fernanda Ballesteros, Laura Barcan, Laura Barcelona, Waldo Belloso, Veronica Berdiñas, Pablo Bonvehi, Juan Pablo Caeiro, Veronica Cisneros, Ana Crinejo, Daniel David, Luz Doldan, Juan Ebenrstejin, Flavio Lipari, Ana Lopardo, Gustavo Lopardo, Marcelo Losso, Pablo Lucchetti, Sergio Lupo, Laura Moreno Macias, Alejandra Moricz de Tesco, Analia Mykietiuk, Estaban Nannini, Gabriel Nieto, Laura Nieto, Luciana Peroni, Ignacio Retta, Patricia Rodriguez, Marisa Sanchez, Pablo Sanchez, Mariana de Paz Sierra, Silvina Tavella, Elena Temporiti, Liliana Trape, Ines Vieni, Eduardo Warley, Diego Yahni, Abel Humberto Zarate.

Thailand (n = 895): Anchalee Avihingsanon, Kanlaya Charoentonpuban, Ploenchan Chetchotisakd, Peeraporn Kaewon, Naphassanant Laopraynak, Weerawat Manosuthi, Kanitta Pussadee, Opass Putcharoen, Kiat Ruxrungtham, Gompol Suwanpimonkul, Sasiwimol Ubolyam.

United States (n = 654): Roberto Arduino, Barbara Atkinson, Taryn M. Aulicino, Jason V. Baker, Cindy Bardascino, Caitlin Bass, John D. Baxter, Mark Beilke, Beverly D. Bentley, Mary Lee Bertrand, Ann B. Brown, June Carbonneau, Richard Cindrich, Patty Coburn, Calvin J. Cohen, Linda Clark, Shirley Cummins, Paul Dassow, Jack A. DeHovitz, Nila J. Dharan, Leslie Faber, Marti Farrough, Matthew Freiberg, Edward Gardner, Kimberly Jo Garrett, Christiane Geisler, Marshall Glesby, Julia Green, Joanne Grenade, Edie Gunderson, John Gunter, Kirsis Ham, Susan Holman, Valery Hughes, Christopher Hurt, Mary Johnson, Glory Koerbel, Susan Koletar, Audrey Lan, Rodger MacArthur, Cheryl Marcus, Norm Markowitz, Maria Laura Martinez, Karen McLaughlin, Raquel Nahra, Mary Jane Nettles, Daniel Nixon, Richard Novak, Kathleen Nuffer, Hannah B. Olivet, Bola Omotosho, Armando P. Paez, Marta Paez-Quinde, Sonija Parker, Namrata Patil, Hari Polenakovik, Sandra Powell, Rachel A. Prosser, Nancy A. Reilly, Paul F. Riska, Stacey Rizza, Robert Schooley, Marla Schwarber, James Scott, Gary L. Simon, Jon Sivoravong, Daniel J. Skiest, Clemencia Solorzano, Rita Sondengam, Nicole Swanson, Ellen Tedaldi, Zelalem Temesgen, Doug Thomas, Bill Thron, Colleen Traverse, David E. Uddin, Daniel Z. Uslan, Marina Vasco, William M. Vaughan, Isabel Vecino, Barbara Wade, Catrice Walker, Kathy Watson, Vicky Watson, David Wohl, Cameron R. Wolfe.

Belgium (n = 549): Leslie Andry, Mireille Bielen, Nathan Clumeck, Eric Florence, Kabamba Kabeya, Jolanthe Sagaer, Jozef Weckx.

Greece (n = 232): Olga Anagnostou, Anastasia Antoniadou, George Daikos, Vicky Gioukari, Ioannis Kalomenidis, Maria Kantzanou, Georgios Koratzanis, Nikolaos Koulouris, Efstratios Maltezos, Symeon Metallidis, Vlassis Polixronopoulos, Helen Sambatakou, Athanasios Skoutelis, Giota Touloumi, Nikolaos Vasilopoulos.

Australia (n = 229): Mark Bloch, Nicky Cunningham, Dominic E. Dwyer, Sian Edwards, Julian Elliott, Jill Garlick, Philip Habel, Fiona Kilkenny, Helen Lau, Karen MacRae, John McBride, Richard Moore, Isabel Prone, Ristila Ram, Sue Richmond, Norm Roth, Tuck Meng Soo, Jo-Anne Thompson, Trina Vincent, Emanuel Vlakahis, Rachel Woolstencroft.

United Kingdom (n = 199): David Chadwick, Tristan Clarke, Jane Democratis, David Dockrell, Robert Heyderman, Ben Jeffs, Stefan Kutter, Martin Llewelyn, Jane Minton, Melanie Newport, Ashley Price.

Peru (n = 193): Carlos Benites, Raul Castillo, Romina Chinchay, Eva Cornelio, Maria Guevara, Luis Gutierrez, Jose Hidalgo, Alberto La Rosa, Yvett Pinedo, Maria Saenz, Juan Vega.

Denmark (n = 180): Bente Baadegaard, Karen Bach, Philippa Collins, Jan Gerstoft, Lene Hergens, Lene Pors Jensen, Zillah Maria Joensen, Gitte Kronborg, Iben Rose Loftheim, Henrik Nielsen, Lars Oestergaard, Court Pedersen, Jens Aage Stauning, Svend Stenvang Pedersen, Yordanos Yehdego.

Germany (n = 170): Frank Bergmann, Christoph Boesecke, Johannes R. Bogner, Norbert Brockmeyer, Christine Czaja-Harder, Rika Draenert, Gerd Fätkenheuer, Hartwig Klinker, Tim Kümmerle, Clara Lehmann, Vera Müller, Andreas Plettenberg, Jürgen Rockstroh, Stefan Schlabe, Wolfgang E. Schmidt, Dirk Schürmann, Gundolf Schüttfort, Ulrich Seybold, Christoph Stephan, Albrecht Stoehr, Klaus Tillmann, Susanne Wiebecke, Timo Wolf.

Spain (n = 163): Jose Arribas, Javier Carbone, Eduardo Fernández Cruz, David Dalmau, Vincente Estrada, Patricia Herrero, Hernando Knobel, Paco López, Rocío Montejano, José Sans Moreno, José Ramón Paño, Begoña Portas, Maria Rodrigo, Pilar Romero, Domingo Sánchez-Sendín, Vincente Soriano.

Poland (n = 130): Elzbieta Bakowska, Andrzej Jerzy Horban, Brygida Knysz, Karolina Pyziak Kowalska, Anna Zubkiewicz-Zarebska.

Estonia (n = 99): Kerstin Kase, Helen Mülle, Kai Zilmer.

Chile (n = 24): Gladys Allendes, Jimena Flores, Rebeka Northland, Carlos Perez, Isabel Velasco, Marcelo Wolff.

China (n = 24): Man-Yee Chu, Tak-chiu Wu.

Austria (n = 20): Heinz Burgmann, Selma Tobudic.

Japan (n = 14): Mayumi Imahashi, Junji Imamura, Yasumasa Iwatani, Ayumi Kogure, Masashi Nakahata, Wataru Sugiura, Yoshiyuki Yokomaku.

Norway (n = 8): Anne Maagaard.

1. Auton A, Abecasis GR, Altshuler DM, et al. A global reference for human genetic variation. Nature. **2015**; 526(7571):68–74.

2. Chang CC, Chow CC, Tellier LCAM, Vattikuti S, Purcell SM, Lee JJ. Second-generation PLINK: Rising to the challenge of larger and richer datasets. Gigascience. **2015**; 4(1):1–16.

3. Delaneau O, Zagury JF, Marchini J. Improved whole-chromosome phasing for disease and population genetic studies. Nat Methods. Nature Publishing Group; **2013**; 10(1):5–6.

4. Alexander DH, Novembre J, Lange K. Fast model-based estimation of ancestry in unrelated individuals. Genome Res. **2009**; 19(9):1655–1664.

5. D. Turner S. qqman: an R package for visualizing GWAS results using Q-Q and manhattan plots. J Open Source Softw. **2018**; 3(25):731.

6. Juliusdottir T. topr: an R package for viewing and annotating genetic association results. BMC Bioinformatics. **2023**; 24(1):268.

7. R Core Team. R: A language and environment for statistical computing. Vienna: R Foundation for Statistical Computing; 2021.

8. Pairo-Castineira E, Rawlik K, Bretherick AD, et al. GWAS and meta-analysis identifies 49 genetic variants underlying critical COVID-19. Nature. **2023**; 617(7962):764-768.

9. Allen EK, Randolph AG, Bhangale T, et al. SNP-mediated disruption of CTCF binding at the IFITM3 promoter is associated with severe influenza risk in humans. Nat Med. **2018**; 23(8):975–983.

10. Pairo-Castineira E, Rawlik K, Bretherick AD, et al. GWAS and meta-analysis identifies 49 genetic variants underlying critical COVID-19. Nature [Internet]. **2023**; 617(7962):764–768. Available from: https://doi.org/10.1038/s41586-023-06034-3
